# TNF-induced necroptosis and PARP-1-mediated necrosis represent distinct routes to programmed necrotic cell death

**DOI:** 10.1007/s00018-013-1381-6

**Published:** 2013-06-13

**Authors:** Justyna Sosna, Susann Voigt, Sabine Mathieu, Arne Lange, Lutz Thon, Parvin Davarnia, Thomas Herdegen, Andreas Linkermann, Andrea Rittger, Francis Ka-Ming Chan, Dieter Kabelitz, Stefan Schütze, Dieter Adam

**Affiliations:** 1 Institut für Immunologie, Christian-Albrechts-Universität zu Kiel, Michaelisstr. 5, 24105 Kiel, Germany; 2 Institut für Klinische und Experimentelle Pharmakologie, Christian-Albrechts-Universität zu Kiel, Hospitalstr. 4, 24105 Kiel, Germany; 3 Klinik für Innere Medizin IV, Nieren- und Hochdruckkrankheiten, Christian-Albrechts-Universität zu Kiel, Schittenhelmstr. 12, 24105 Kiel, Germany; 4 Biochemisches Institut, Christian-Albrechts-Universität zu Kiel, Olshausenstr. 40, 24098 Kiel, Germany; 5 Department of Pathology, University of Massachusetts Medical School, 55 Lake Avenue North, Worcester, MA 01655 USA; 6 Present Address: Ferring GmbH, Wittland 11, 24109 Kiel, Germany

**Keywords:** Necroptosis, Programmed necrosis, TNF, PARP-1

## Abstract

Programmed necrosis is important in many (patho)physiological settings. For specific therapeutic intervention, however, a better knowledge is required whether necrosis occurs through one single “core program” or through several independent pathways. Previously, the poly(ADP-ribose) polymerase (PARP) pathway has been suggested as a crucial element of tumor necrosis factor (TNF)-mediated necroptosis. Here, we show that TNF-induced necroptosis and the PARP pathway represent distinct and independent routes to programmed necrosis. First, DNA-alkylating agents such as 1-methyl-3-nitro-1-nitrosoguanidine (MNNG) or methyl methanesulfonate rapidly activate the PARP pathway, whereas this is a late and secondary event in TNF-induced necroptosis. Second, inhibition of the PARP pathway does not protect against TNF-induced necroptosis, e.g., the PARP-1 inhibitor 3-AB prevented MNNG- but not TNF-induced adenosine-5′-triposphate depletion, translocation of apoptosis-inducing factor, and necrosis. Likewise, olaparib, a more potent and selective PARP-1 inhibitor failed to block TNF-induced necroptosis, identical to knockdown/knockout of PARP-1, pharmacologic and genetic interference with c-Jun N-terminal kinases and calpain/cathepsin proteases as further components of the PARP pathway. Third, interruption of TNF-induced necroptosis by interference with ceramide generation, RIP1 or RIP3 function or by the radical scavenger butylated hydroxyanisole did not prevent programmed necrosis through the PARP pathway. In summary, our results suggest that the currently established role of the PARP pathway in TNF-induced necroptosis needs to be revised, with consequences for the design of future therapeutic strategies.

**Electronic supplementary material:**

The online version of this article (doi:10.1007/s00018-013-1381-6) contains supplementary material, which is available to authorized users.

## Introduction

Programmed cell death (PCD) is essential for survival and homeostasis of multicellular organisms. Whereas induction of PCD through activation of caspases (subsequently termed “apoptosis”) represents the primary route to PCD in most physiological settings, alternative forms of PCD have been discovered that operate without detectable caspase activity. Of those, programmed necrosis represents the main mode of caspase-independent PCD [1]. Programmed necrosis can participate in vital processes when caspase-dependent pathways are abrogated, for example in the removal of interdigital webs during embryogenesis in Apaf-1-deficient embryos [2]. Furthermore, programmed necrosis contributes to the negative selection of lymphocytes, termination of immune responses, regulation of bone growth, ovulation, cellular turnover in the intestine or post-lactational regression of the mammary gland [2–4]. In addition, programmed necrosis has been implicated in the pathology of hyperacute shock [5], pancreatitis [6, 7], renal [8], cerebral and myocardial ischemia–reperfusion injury, epilepsy, muscular dystrophy, Alzheimer’s, Huntington’s and Parkinson’s disease, as well as in the destruction of cells by pathogens such as HIV, vaccinia virus,* Shigella* and* Salmonella* [1, 2, 9, 10]. As a backup mechanism, programmed necrosis can permit a cell to commit suicide if the caspase machinery fails or is inactivated (e.g., after virus infection or in apoptosis-resistant tumor cells) [2, 11].

Necroptosis represents a subset of programmed necrosis, which is elicited through death receptors and which depends on the activity of the protein kinases RIP1 and RIP3 [6, 7, 10, 11]. Downstream of RIP3, the very recently discovered proteins MLKL and PGAM5 further convey the necroptotic signal by inducing mitochondrial fragmentation [12]. Enzymes of the energy metabolism and production of reactive oxygen species (ROS), e.g., by the NADPH oxidase Nox1 or by mitochondria, as well the deubiquitinase CYLD and the Bcl-2 family member Bmf represent further candidate mediators of necroptosis [1]. Moreover, FADD and caspase-8 constitute essential negative regulators of RIP1/RIP3-mediated necroptosis during normal embryonic development [11].

The two most extensively studied models of programmed necrosis are necroptosis initiated by the 55-kDa tumor necrosis factor (TNF) receptor (TNF-R1) and necrosis mediated via the poly(ADP-ribose) polymerase (PARP) pathway. The latter is initiated by overactivation of the DNA repair enzyme PARP-1, e.g., in response to DNA-alkylating agents such as 1-methyl-3-nitro-1-nitrosoguanidine (MNNG), resulting in the massive synthesis of poly(ADP-ribose) (PAR) from nicotinamide adenine dinucleotide (NAD^+^) and, in consequence, to the rapid depletion of intracellular NAD^+^ and adenosine-5′-triposphate (ATP) pools. Subsequently, and involving c-Jun N-terminal kinases (JNK), the mitochondrial protein Bax and calpain/cathepsin proteases, cleavage of apoptosis-inducing factor (AIF) occurs, which then translocates from mitochondria to the cytosol and further to the nucleus where it forms an active DNA-degrading complex with histone H2AX and cyclophilin A. This complex then induces caspase-independent, large-scale DNA fragmentation and finally, necrosis [1, 13, 14].

A previous study has implicated the PARP pathway as an integral part of TNF-induced necroptosis in L929 fibrosarcoma cells [15]. Since the existence of one core program of necroptosis would allow a more efficient therapeutic interference than the existence of several distinct pathways that would have to be targeted simultaneously, this would also have implications for the future development of antinecrotic cytoprotective drugs [1].

We have previously demonstrated that the sphingolipid ceramide represents a key mediator of TNF-induced necroptosis in L929 cells as well as in other cell systems [16, 17]. In our efforts to more precisely define the targets of ceramide in TNF-induced necroptosis, we extended our investigations to components of the PARP pathway. Unexpectedly, our results indicate that the PARP pathway is not integral to TNF-induced necroptosis, but rather that both pathways represent distinct and independent routes to programmed necrosis.

## Materials and methods

*Reagents.* Highly purified human recombinant TNF was provided by BASF Bioresearch (Ludwigshafen, Germany). Methyl methanesulfonate (MMS), cycloheximide (CHX), benzyloxycarbonyl-Phe-Ala-fluoromethylketone (zFA-fmk), *trans*-epoxysuccinyl-l-leucylamido(4-guanidino)butane (E-64), pepstatin A, imipramine and butylated hydroxyanisole (BHA) were purchased from Sigma (Deisenhofen, Germany), MNNG either from Sigma or from ABCR (Karlsruhe, Germany). Carbobenzoyl-l-leucyl-l-leucyl-l-leucinal (MG-132) and poly(ADP-ribose) glycohydrolase (PARG) purified from bovine thymus were from Enzo (Lausen, Switzerland). 3-aminobenzamide (3-AB) and N-(5,6-dihydro-6-oxo-2-phenanthridinyl)-2-acetamide hydrochloride (PJ-34) were obtained from TCI (Eschborn, Germany) and from Tocris (Ellisville, MO, USA), olaparib was purchased from Axon (Groningen, The Netherlands). Benzyloxycarbonyl-Val-Ala-Asp(OMe)-fluoromethylketone (zVAD) was from Bachem (Bubendorf, Switzerland), JNK-inhibitor III and necrostatin-1 were obtained from Merck (Darmstadt, Germany) and N-[l-3-*trans*-(propylcarbamoyl)-oxirane-2-carbonyl]-l-Ile-l-Pro methyl ester (CA-074 Me) from Biomol (Hamburg, Germany).

*Cell culture.* L929ATCC, HeLa, wild-type Jurkat, HT-29, 293T, and MCF-7 cells were obtained from American Type Culture Collection (ATCC, Manassas, VA, USA). L929sA cells [18] were kindly provided by Peter Vandenabeele (Ghent, Belgium). L929Ts is a tumor necrosis factor-related apoptosis-inducing ligand (TRAIL)-sensitive L929 subline derived in our laboratory [19]. Wild-type and PARP-1-deficient mouse embryonic fibroblasts (MEF) were kindly provided by Françoise Dantzer (Illkirch, France). RIP1-deficient Jurkat cells were a gift from Brian Seed (Boston, MA, USA). Wild-type and RIP1-deficient MEF have been previously described [20], as have NIH3T3 cells naturally expressing or deficient for RIP3 [7]. NIH3T3 transfectants stably expressing murine green-fluorescent-protein (GFP) fusions to wild-type or kinase-defective murine RIP3 in the vector pEGFP-N1 (Takara, Mountain View, CA, USA) were generated by selection in DMEM medium containing 1 mg/ml G418 (PAA, Cölbe, Germany). Positive clones were identified by flow cytometry. Primary lung fibroblasts from JNK1-, JNK2-, and RIP3-deficient mice and their wild-type littermate controls were prepared and cultured as described [17]. Immortalized wild-type and JNK1/JNK2 double-deficient MEF were a kind gift from Roger Davis (Worcester, MA, USA). Wild-type, cathepsin D-deficient, and cathepsin D-deficient MEF stably transfected with the pro-cathepsin D cDNA have been previously described [21], as have TNF-R1/R2 double-deficient mouse fibroblasts and their transfectants stably re-expressing TNF-R1 [22]. Wild-type and RIP3-deficient MEF were generated from d14.5 embryos according to standard procedures. Cells were cultivated in DMEM (NIH3T3, HeLa, MEF), McCoy’s (HT-29), RPMI 1640 (293T, MCF-7, TNF-R1/2 double-deficient mouse fibroblasts) or a mixture of Click’s/RPMI 1640 medium (all other cell lines) supplemented with 10 % v/v calf serum and 10 mM glutamine in a humidified incubator containing 5 % w/v CO_2_. Media were additionally supplemented with 1 mM sodium pyruvate (HT-29, 293T, MCF7) and 50 μg/ml each of streptomycin and penicillin (except for 293T and MCF-7 cells).

*Immunoblots.* Unless otherwise indicated, cells were harvested after treatment and lysed at 4 °C in TNE buffer (50 mM Tris pH 8.0, 1 % v/v NP40, 2 mM EDTA) containing 10 μg/ml pepstatin/aprotinin/leupeptin, 1 mM sodium orthovanadate and 5 mM NaF. AIF was detected in purified cytosolic fractions generated with the ProteoJET (Fermentas, St. Leon-Rot, Germany) nuclear and cytoplasmic extraction kit. Identical amounts of cell protein per lane were resolved by electrophoresis on SDS polyacrylamide gels. After electrophoretic transfer to nitrocellulose, reactive proteins were detected using antisera specific for actin (sc-1615, Santa Cruz, Heidelberg, Germany; A1978, Sigma), PARP-1 (556362, Becton–Dickinson, Heidelberg, Germany; 9542, Cell Signaling, Danvers, MA, USA), TRADD (3684, Cell Signaling), FADD (7B5, Enzo), Caspase-8 (4927, Cell Signaling), RIP1 (610458, Becton–Dickinson), PAR (551813, Becton–Dickinson), AIF (sc-13116, Santa Cruz), COXIV (sc-58348, Santa Cruz), histone (ab1791, Abcam, Cambridge, UK), JNK1 (554286, Becton–Dickinson), JNK2 (sc-271133, Santa Cruz), phospho-JNK (AF1205, R&D Systems, Wiesbaden, Germany), TNF-R1 (sc-8436, Santa Cruz), human (PAB0287, Abnova, Heidelberg, Germany) or murine RIP3 (ADI-905-242, Enzo), GFP (8369-1, Takara), and the ECL detection kit (GE Healthcare, Munich, Germany). Equal loading as well as efficiency of transfer was routinely verified for all Western blots by Ponceau S staining, and by reprobing the membranes for actin.

*Quantitative real*-*time polymerase chain reaction (qRT*-*PCR)*. Total RNA was isolated from L929Ts cells after induction of necroptosis with 100 ng/ml TNF for 0, 10, 15 and 20 h using the High Pure RNA isolation kit (Roche, Mannheim, Germany) and transcribed into single-stranded cDNA using the 1st Strand cDNA Synthesis Kit for RT-PCR (AMV, Roche) following the recommendations of the manufacturer. Quantification of PARP-1 expression levels was carried out with primers 5′-ATGCTACCACGCACAAC-3′ and 5′-CCAATCGGGTCTCCCT-3′ for murine PARP-1 with murine glyceraldehyde 3-phosphate dehydrogenase (primers 5′-ACCACAGTCCATGCCATCAC-3′ and 5′-TCCACCACCCTGTTGCTGTA-3′) as internal standard, using a LightCycler System, the LightCycler–FastStart DNA Master SYBR Green I Kit and LightCycler software version 3.5 (Roche).

*Measurement of intracellular NAD* ^+^ *and ATP levels*. Cytosolic extracts were prepared in a buffer containing 0.5 % v/v Triton X-100, 10 mM Tris pH 7.5, and 1 mM EDTA. For measurement of NAD^+^, identical amounts of protein were incubated for 30 min at 37 °C with 100 μl assay buffer (8 % v/v ethanol, 1 % w/v polyvinylpyrrolidone, 0.5 mM EGTA, 50 mM Tris/HCl pH 8.5, 1 mM phenazine ethosulfate, 0.44 mM 3-(4,5-dimethylthiazol-2-yl)-2,5-diphenyltetrazolium bromide, 2 U alcohol dehydrogenase) before absorption was determined at 570 nm in a microplate reader (Tecan, Crailsheim, Germany). The cellular ATP content of the lysates was determined using a bioluminescence assay according to the manufacturer’s instructions (FL-AA, Sigma) in a FB-12 luminometer (Berthold, Bad Wildbad, Germany). Alternatively, intracellular ATP levels were determined with the Cell Titer Glo Assay Kit (Promega, Mannheim, Germany).

*Flow cytometric analysis of membrane integrity.* Cells were seeded in 12-well plates at 5 × 10^4^ cells/well. Following treatment, both detached and adherent cells were collected by centrifugation. The cells were resuspended in PBS/5 mM EDTA containing 2 μg/ml propidium iodide (PI), and the red fluorescence was measured on a FACSCalibur flow cytometer (Becton–Dickinson).

*RNA interference.* The validated siRNA specific for human PARP1 (ID # s1097), the predesigned siRNA specific for murine PARP-1 (ID # s62053), the custom-made siRNA specific for murine RIP1 (target sequence 5′-CCACUAGUCUGACUGAUG-3′), the predesigned siRNA specific for murine RIP3 (ID # s80755) as well as the negative control siRNA were obtained from Life Technologies, Darmstadt, Germany. L929Ts and Jurkat cells were transfected with 150 pmol siRNA by Amaxa nucleofection (Lonza, Cologne, Germany), using solution V and program X-001, and incubated for 72 h at 37 °C before further analysis. NIH3T3 cells naturally expressing RIP3 were transfected as above, except for using solution R and program A-24, and incubation for 48 h.

## Results

### TNF and MNNG activate PARP-1, but with distinct kinetics

In L929 cells, TNF elicits PCD exclusively by necroptosis but not by apoptosis [16, 17, 23]. Likewise, MNNG kills cells by necrosis rather than by apoptosis [14, 24]. Accordingly, three L929 cell lines available in our laboratory (TRAIL-sensitive L929Ts cells, “genuine” L929ATCC cells freshly obtained from ATCC and TNF-hypersensitive L929sA cells) did not display caspase activation or apoptotic membrane blebbing after treatment with TNF or MNNG (unlike TNF/CHX-treated positive controls, Supplementary Figure S1), confirming that in the L929 cell lines utilized for this study, both MNNG and TNF induce cell death through necrotic pathways rather than through caspase-dependent apoptosis.

**Fig. 1:**
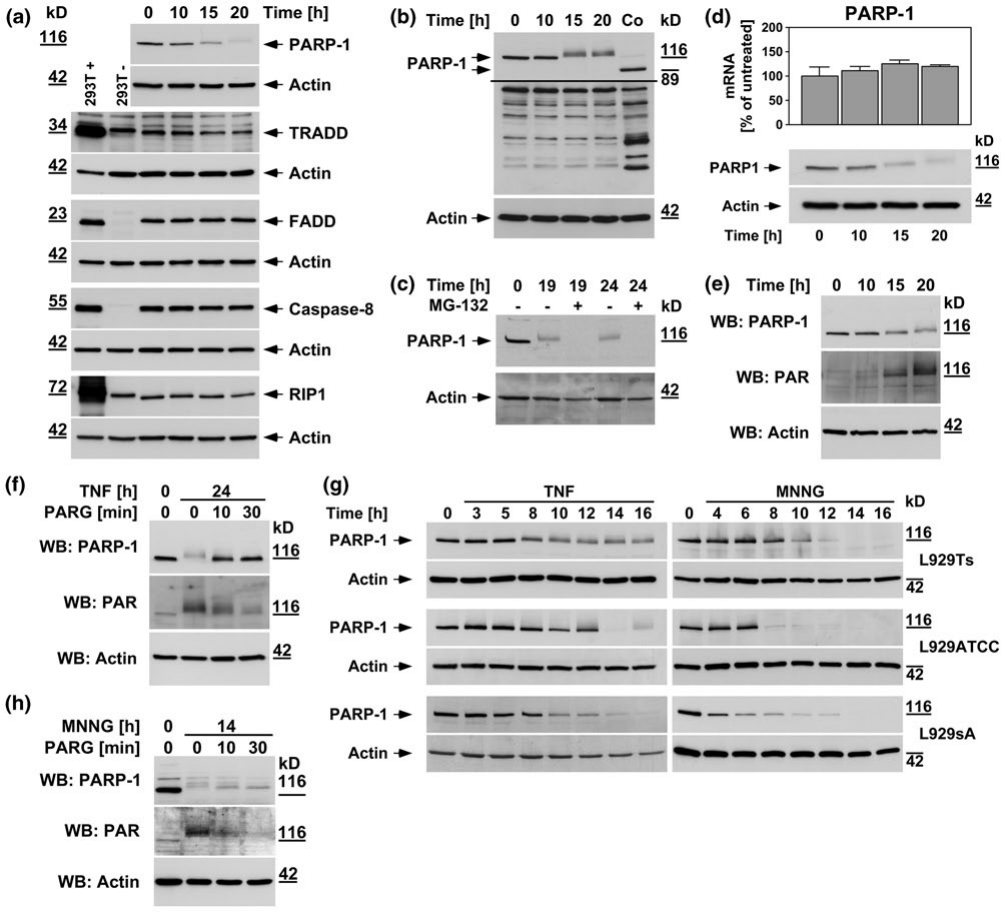
PARP-1 is activated with distinct kinetics by TNF and MNNG. In (**a**–**f**), L929Ts cells were treated with 100 ng/ml TNF for the indicated times. **a** Cells were analyzed for PARP-1 by immunoblotting with antibody 9542. The same lysates were additionally analyzed on separate gels for TRADD, FADD, caspase-8, and RIP1.* Arrows* indicate the positions of the full-length proteins, which were additionally verified for TRADD, FADD, caspase-8, and RIP1 by parallel separation of lysates from 293T cells overexpressing the respective protein (293T+). Untransfected 293T cells (293T−) were included for control. Shifted signals for TRADD and RIP1 in these lanes represent the cross-reactive endogenous human proteins that differ in size from the endogenous murine proteins of L929Ts cells. **b** Western blot with antibody 9542 that detects all cleavage fragments of PARP-1. Arrows indicate the position of the full-length protein and the 89-kDa caspase-3 cleavage product present only in apoptotic control lysates (Co, extracts from cells treated with 100 ng/ml TNF and 2 μg/ml of the protein biosynthesis inhibitor CHX for 1 h). A longer exposure is shown for the lower part of the blot to increase the visibility of the reactive bands. **c** Cells were additionally treated with the proteasome inhibitor MG-132 (52.6 μM) as indicated. PARP-1 was detected with antibody 556362. **d** The expression levels of PARP-1 mRNA were determined by qRT-PCR in parallel to protein levels (shown below, PARP-1 was detected with antibody 9542). The expression of PARP-1 is shown relative to untreated cells. Comparable results were obtained with a different primer pair. **e** Following detection of PARP-1 (antibody 9542), the membrane was reprobed with an antibody for PAR to reveal the presence of poly(ADP-ribosyl)ated proteins. **f** Lysates from cells treated with TNF for the indicated times were additionally incubated for the indicated times in the presence of 10 mM β-mercaptoethanol and 0.05 U PARG before PARP-1 was detected with antibody 9542 and reprobing of the membrane with an antibody for PAR. **g** L929Ts, L929ATCC, and L929sA cells were left untreated, treated with TNF as above, or treated with 0.5 mM MNNG for 15 min and further incubated with fresh medium without MNNG for the indicated times before PARP-1 was detected with antibody 9542. **h** Lysates from L929Ts cells treated with MNNG as in (**g**) for the indicated times were incubated with PARG and analyzed as in (**f**). For all Western blot panels, detection of actin served as a loading control. For all figures, representative data from one out of at least two or more experiments with similar results are shown (*n* ≥ 2) and *error bars* indicate the standard deviations (SD) from at least triplicate determinations (*n* ≥ 3)

During apoptosis, PARP-1 is inactivated by caspase-3-mediated cleavage of the mature 116-kDa protein to an 89-kDa product [16]. However, in L929Ts cells undergoing TNF-induced necroptosis, PARP-1 displays an atypical size shift/disappearance of the mature, uncleaved protein within 20 h (Fig. 1a) [16, 19]. We have previously shown that caspases are not responsible for this process [16]. This disappearance is also not due to a general necroptotic destruction of cellular proteins, since TRADD, FADD, caspase-8, or RIP-1 did not show such changes in their levels (Fig. 1a). An PARP-1 antibody recognizing all cleavage fragments detected the apoptotic 89-kDa fragment in control extracts from L929Ts cells (Fig. 1b, Co), together with a pattern of additional bands. However, this pattern did not change in TNF-induced necroptosis (except for the disappearance of full-length PARP-1), and no additional cleavage products of PARP-1 were detected (Fig. 1b). Since we observed a time-dependent shift of the mature PARP-1 signal to higher molecular weights (Fig. 1a–g), we speculated that ubiquitylation [25] followed by complete proteasomal degradation might cause the disappearance of PARP-1. Treatment with the proteasome inhibitor MG-132 did not however prevent but rather accelerated the disappearance of the PARP-1 signal (Fig. 1c). Similarly, we could rule out that a downregulation of PARP-1 at the transcriptional level is responsible for its disappearance (Fig. 1d). Rather, in extracts from TNF-treated L929Ts cells, an antibody against PAR showed reactivity that co-migrated with the PARP-1 signal, and concurrently shifted to higher molecular weights in the course of necroptosis (Fig. 1e). Since the most prominent target protein of PARP-1 poly(ADP-ribosyl)ation is PARP-1 itself [26], we hypothesized that the disappearance of the PARP-1 signal in Western blots during TNF-induced necroptosis was due to heavy poly(ADP-ribosyl)ation, rendering PARP-1 inaccessible for detection by PARP-1 antibodies. Confirming our assumption, we could revert both disappearance and size shift as well as the associated poly(ADP-ribosyl)ation of PARP-1 by in vitro addition of PARG to cell lysates (Fig. 1f). Therefore, the disappearance of the PARP-1 signal is an indicator of PARP-1 activation rather than PARP-1 destruction.

We confirmed the TNF-dependent activation/disappearance of PARP-1 in time-course analyses not only in L929Ts but also in L929ATCC and L929sA cells (Fig. 1g). In the same three cell lines, the potent PARP1 activator MNNG [24] likewise induced the disappearance of PARP-1, however more rapidly, with changes already detectable within 4–8 h after treatment (Fig. 1g). For TNF, this occurred at 10 h of treatment or later, indicating that the initial steps of TNF-induced necroptosis do not require activation of PARP-1. Furthermore, in vitro addition of PARG reverted disappearance, size shift, and poly(ADP-ribosyl)ation of PARP-1 also in lysates from MNNG-treated L929Ts cells (Fig. 1h), confirming that the disappearance of PARP-1 in response to MNNG is likewise due to activation and auto-poly(ADP-ribosyl)ation of PARP-1 itself (as independently reported previously [14, 27, 28]).

### DNA-alkylating agents but not TNF cause rapid activation of the PARP pathway

**Fig. 2:**
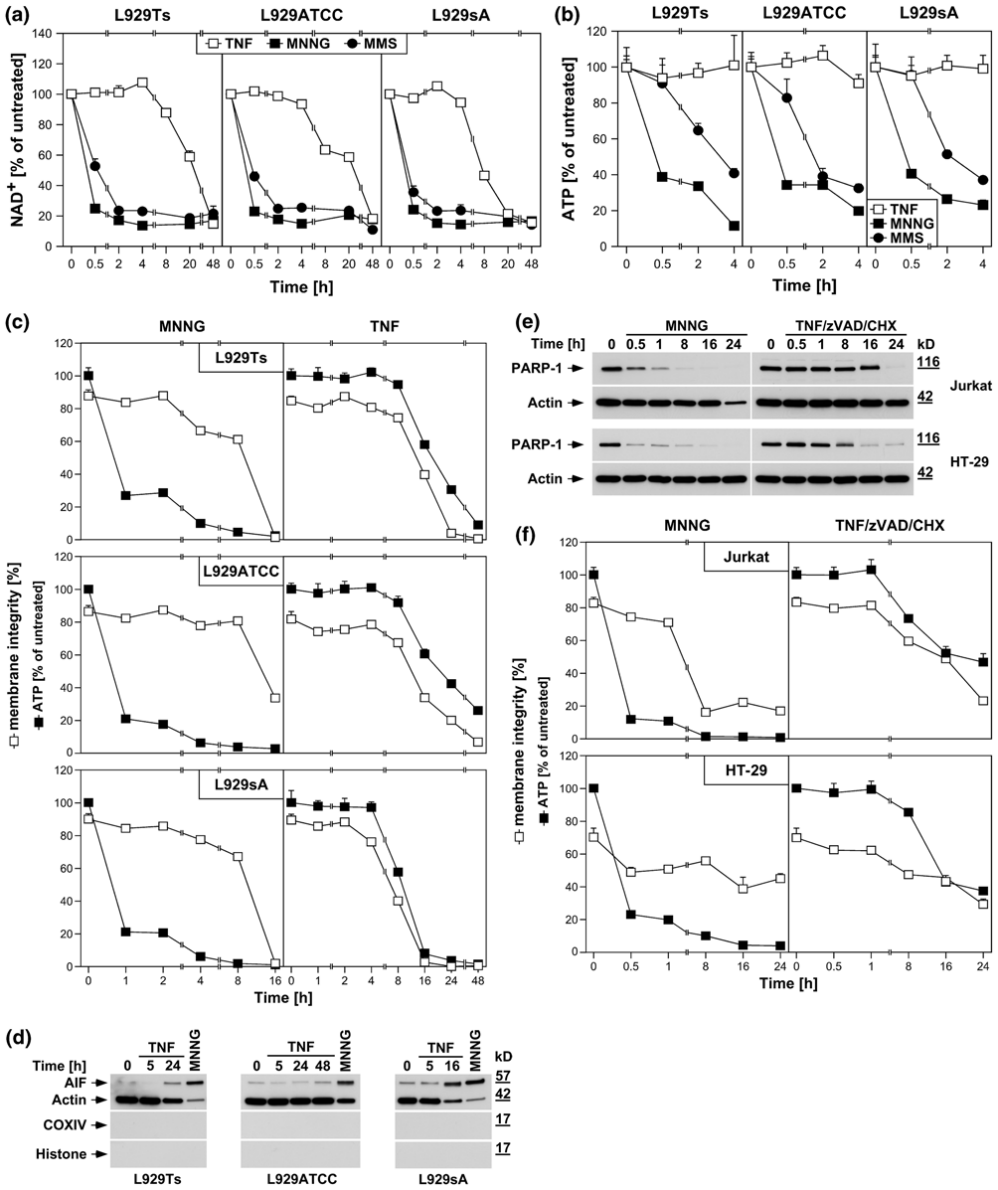
DNA-alkylating agents but not TNF activate the PARP pathway. **a** L929ATCC, L929Ts, and L929sA cells were stimulated with 100 ng/ml TNF for the indicated times, or with 0.5 mM MNNG or 25 mM MMS (as a distinct DNA-alkylating agent) for 15 min and subsequent incubation with fresh medium without MNNG/MMS for the indicated times. Subsequently, their intracellular NAD^+^ content was measured and is shown relative to untreated cells. **b** Cells were stimulated as in (**a**) and intracellular ATP levels were measured. **c** L929ATCC, L929Ts, and L929sA cells were stimulated with MNNG or TNF as in (**a**) and analyzed for intracellular ATP content and loss of membrane integrity (as a marker for cell death) at the indicated time points. Membrane integrity was measured by PI staining and flow cytometry. For clarity of presentation, the percentage of intact, large PI-negative cells that still retain their membrane integrity is shown. **d** Purified cytosolic fractions from L929Ts, L929ATCC, and L929sA cells were analyzed for translocation of AIF into the cytosol following stimulation with 100 ng/ml TNF for the indicated times or with 1 mM MNNG for 15 min and subsequent incubation with fresh medium without MNNG for 14 h. The purity of the fractions was verified by detection of actin (cytosolic marker), COXIV (mitochondrial marker) and histone (nuclear marker). Equal loading was verified by staining with Ponceau S (Supplementary Figure S2a), since actin displayed a decrease in these highly purified fractions during cell death. The activity of the employed COXIV and histone antibodies was confirmed with positive control lysates (Supplementary Figure S2d). **e** Jurkat and HT-29 cells were stimulated with MNNG as in (**a**) or 100 ng/ml TNF with addition of 20 μM (HT-29) or 50 μM (Jurkat) of the broad-spectrum caspase inhibitor zVAD-fmk to prevent apoptosis and 2 μg/ml (Jurkat) or 5 μg/ml (HT-29) CHX to sensitize the cells [17]. Subsequently, PARP-1 was detected with antibody 9542. Detection of actin served as a loading control. **f** In parallel, the cells were analyzed for intracellular ATP content and loss of membrane integrity as in (**c**). For all flow cytometric analyses of membrane integrity, we measured the percentage out of a total of 10,000 analyzed cells that show loss of membrane integrity (this is calculated as 100 % minus the percentage of intact, large PI-negative cells to account for disintegrated dead cells that have lost their PI staining again due to diffusion)

In PARP-1-mediated necrosis, hyperactivation of PARP-1 results in a rapid depletion of intracellular NAD^+^ [24]. Accordingly, MNNG and MMS, a distinct DNA-alkylating agent and activator of PARP-1 [29], uniformly induced a rapid decrease of intracellular NAD^+^ in all three L929 sublines, leading to almost complete NAD^+^ depletion within 2 h (Fig. 2a). In contrast, TNF failed to induce notable changes in NAD^+^ levels within this timeframe, but caused a decrease of NAD^+^ only at considerably later time points (8–48 h, Fig. 2a).

As a direct result of excessive NAD^+^ consumption by overactivated PARP-1, intracellular ATP is depleted by the cell’s efforts to resynthesize NAD^+^ [1]. In line, both MNNG and MMS caused the depletion of the intracellular ATP stores of L929Ts, L929ATCC, and L929sA cells within 4 h, whereas TNF again did not induce a notable reduction (Fig. 2b). This discrepancy between TNF and DNA-alkylating agents was further corroborated when we compared ATP levels with the progression of cell death. Both MNNG and TNF led to a pronounced reduction of membrane integrity in all three L929 cell lines. Whereas the rapid loss of intracellular ATP induced by MNNG clearly preceded loss of membrane integrity, this occurred at later time points and concurrent with cell death for TNF (Fig. 2c).

Downstream of NAD^+^/ATP depletion, translocation of AIF from mitochondria to the cytosol and to the nucleus is essential in PARP-1-mediated necrosis [1, 28]. In concordance with our above results, MNNG elicited a pronounced translocation of AIF into the cytosol of L929Ts, L929ATCC and L929sA cells, whereas TNF caused only a marginal translocation of AIF in L929Ts and L929ATCC cells (more pronounced in TNF-hypersensitive L929sA cells), again occurring in the late to end rather than the early stages of cell death (24 h in L929Ts cells, 48 h in L929ATCC cells, 16 h in L929sA cells, Fig. 2d).

We additionally analyzed MNNG- and TNF-induced activation/disappearance of PARP-1, depletion of intracellular ATP and loss of membrane integrity in human leukemic Jurkat T cells and human HT-29 colorectal adenocarcinoma cells as two additional established cell systems for necroptosis [6, 17]. As shown in Fig. 2e, f, we obtained essentially the same results as with the three L929 sublines, confirming that the above findings are not limited to L929 cells. In summary, individual components of the PARP pathway are activated only late in TNF-induced necroptosis (in contrast to their rapid activation by DNA-alkylating agents), most likely as a secondary consequence, but not the cause of necroptotic cellular disintegration.

### Inhibition of the PARP pathway does not protect against TNF-induced necroptosis

**Fig. 3:**
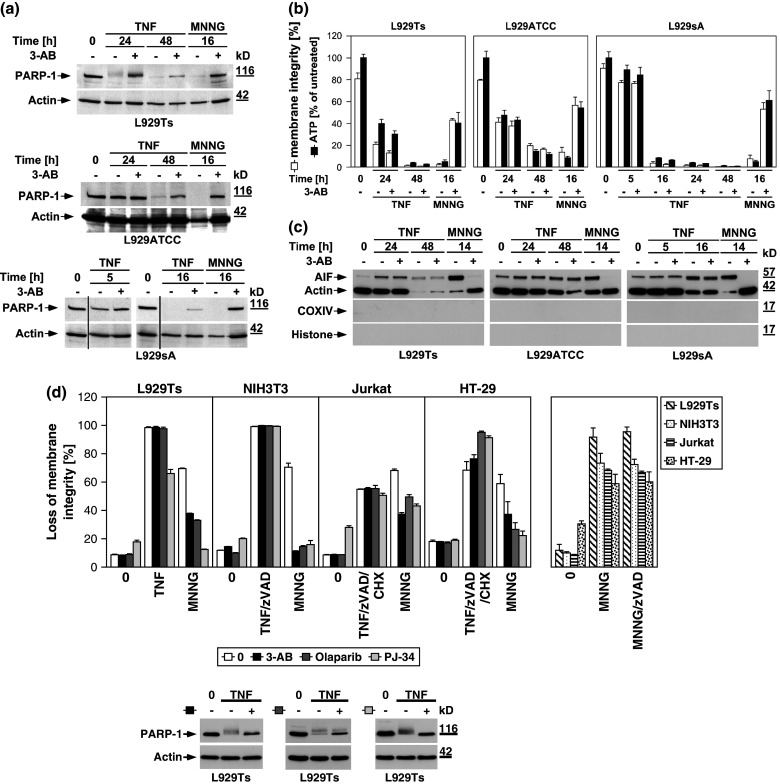

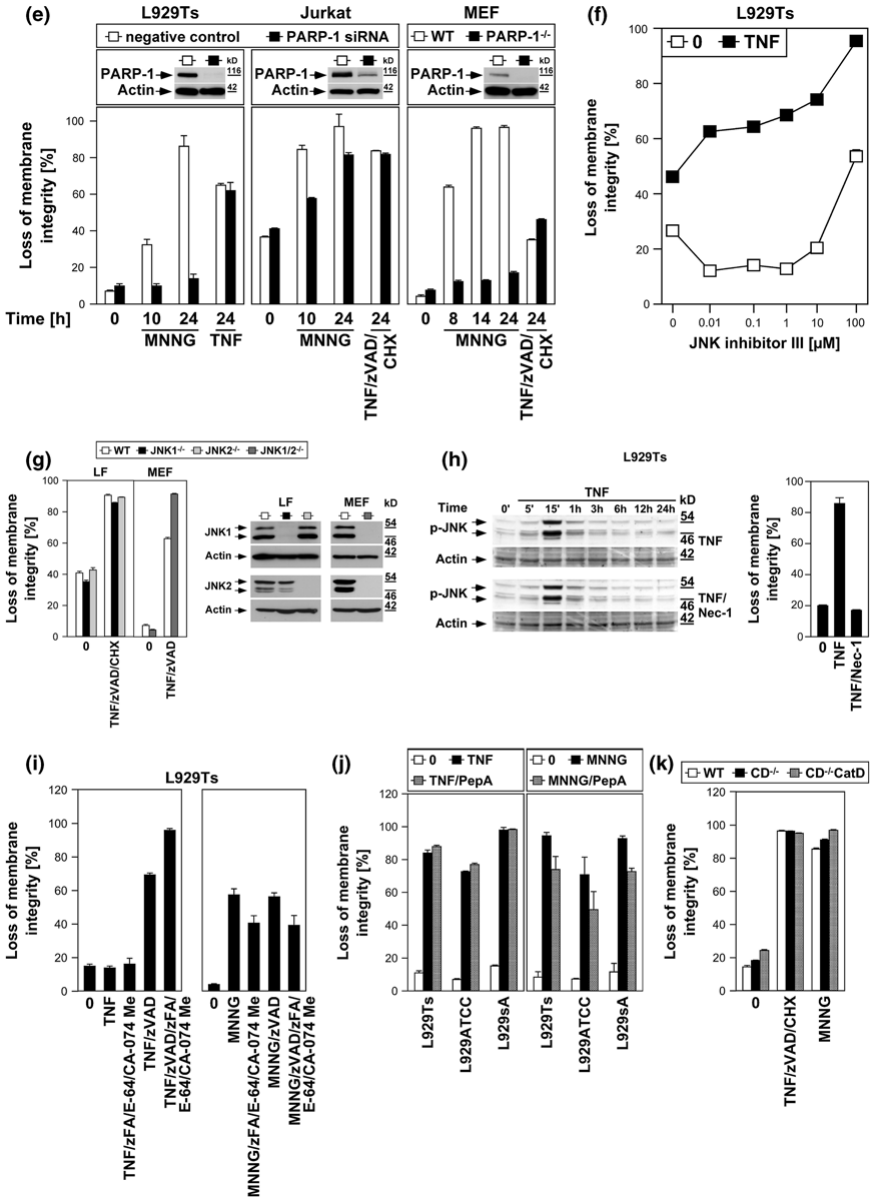
TNF-induced necroptosis is not blocked by inhibition of the PARP pathway. **a** L929Ts, L929ATCC, and L929sA cells were left untreated, treated with 100 ng/ml TNF for the indicated times, or treated with 1 mM MNNG for 15 min and further incubated for 16 h with fresh medium without MNNG in the presence or absence of the PARP inhibitor 3-AB (2.5 mM). Subsequently, activation/disappearance of PARP-1 was detected with antibody 9542. Detection of actin served as a loading control. **b** Cells were stimulated with TNF or with MNNG (0.5 mM) as in (**a**) before intracellular ATP levels were measured and loss of membrane integrity as a marker for cell death was determined by flow cytometry and PI staining. For clarity of presentation, the percentage of intact, large PI-negative cells with preserved membrane integrity is shown. **c** Cells were stimulated as in (**a**) with incubation for 14 h after MNNG treatment before purified cytosolic fractions were analyzed for translocation of AIF. The purity of the fractions was verified by detection of actin (cytosolic marker), COXIV (mitochondrial marker) and histone (nuclear marker). Equal loading was verified by staining with Ponceau S (Supplementary Figure S2b), since actin displayed a decrease in these highly purified fractions during cell death. At 48 h of TNF treatment, L929Ts cells additionally displayed a decrease of AIF, due to this very late stage of cell death. Nevertheless, 3-AB prevented both AIF translocation and actin decrease selectively in MNNG-, but not TNF-treated cells. **d** *Left panels* Necroptosis was induced in L929Ts, NIH3T3, Jurkat, and HT-29 cells in the absence or presence of the PARP1 inhibitors 3-AB (2.5 mM), olaparib (L929: 1 μM; NIH3T3, Jurkat: 50 nM; HT-29: 2 μM) or PJ-34 (20 μM) by treatment with 100 ng/ml TNF for 24 h (L929Ts, HT-29), 16 h (NIH3T3) or 20 h (Jurkat) with addition of 20 μM (NIH3T3, HT-29) or 50 μM (Jurkat) zVAD-fmk and 2 μg/ml (Jurkat) or 5 μg/ml (HT-29) CHX. In parallel, cells were treated with MNNG for 16 h as in (**a**) with or without addition of 3-AB, olaparib or PJ-34. *Right panel* to test whether or not zVAD-fmk inhibits MNNG-induced cell death, cells were treated with MNNG as in (**a**) in the absence or presence of 20 μM (Jurkat: 50 μM) zVAD-fmk. Subsequently, loss of membrane integrity was analyzed by flow cytometry. *Lower panel* To verify the efficacy of the inhibitors (in addition to their effect on MNNG-induced necrosis shown in the *upper panels*), activation/disappearance of PARP-1 in L929Ts cells treated with 100 ng/ml TNF for 24 h in the presence or absence of 2.5 mM 3-AB, 1 μM olaparib or 20 μM PJ-34 cells was detected with antibody 9542. Detection of actin served as a loading control. **e** L929Ts or Jurkat cells were nucleofected with an siRNA that does not elicit an RNA interference response (negative control) or with siRNA specific for murine or human PARP-1 (PARP-1 siRNA). Together with wild-type (WT) and PARP-1-deficient MEF, the cells were stimulated either with 0.5 mM MNNG for 15 min and further incubated for the indicated times with fresh medium without MNNG or with 100 ng/ml TNF in the absence (L929Ts) or presence (Jurkat, MEF) of 20 μM zVAD-fmk and 2 μg/ml (Jurkat) or 1 μg/ml (MEF) CHX for 24 h before loss of membrane integrity was measured by PI staining and flow cytometry. Control Western blots for PARP-1 were performed to verify successful downregulation of endogenous murine or human PARP-1 or to show presence or absence of murine PARP-1. Detection of actin served as a loading control. **f** Inhibition of necroptosis by the JNK-inhibitory peptide JNK-inhibitor III was assayed in L929Ts cells treated with 100 ng/ml TNF for 16 h or not and increasing concentrations of JNK-inhibitor III. **g** *Left panel* Primary lung fibroblasts (LF) from wild-type (WT), JNK1-(JNK1^−/−^) and JNK2-deficient (JNK2^−/−^) mice or immortalized MEF from JNK1/JNK2 double-deficient (JNK1/2^−/−^) mice were left untreated or incubated with 100 ng/ml TNF and 20 μM zVAD-fmk in the presence or absence of 2 μg/ml CHX to elicit necroptosis. After 18 h, loss of membrane integrity was analyzed by flow cytometry. *Right panel* Control Western blots were performed to verify presence or absence of endogenous JNK1 and/or JNK2 and equal loading (actin). **h** L929Ts cells were stimulated with 100 ng/ml TNF for the indicated times with or without the necroptosis inhibitor necrostatin-1 (Nec-1, 10 μM). Activation of JNK was analyzed by detection of phosphorylated p46 JNK1 and p54 JNK2 (*arrows*) in Western blots (*left panel*) using antibody AF1205. Detection of actin served as a loading control. To demonstrate the efficacy of the inhibitor, loss of membrane integrity of identically treated L929Ts cells was determined after 24 h by flow cytometry (*right panel*). **i** L929Ts cells were incubated with 100 ng/ml TNF for 5 h or with MNNG (0.5 mM) as in (**a**) with optional addition of 20 μM zVAD-fmk and/or a combination of 20 μM each of zFA-fmk, E-64, and CA-074 Me before loss of membrane integrity was measured by flow cytometry. **j** L929Ts, L929ATCC and L929sA cells were preincubated for 24 h or not with the cathepsin D inhibitor pepstatin A (PepA, 100 μM) followed by either addition of 100 ng/ml TNF or not for 24 h, or by treatment with MNNG (0.5 mM) as in (**a**) or not. Subsequently, loss of membrane integrity was measured by flow cytometry. **k** Wild-type (WT), cathepsin D-deficient (CD^−/−^), and CD^−/−^ MEF stably transfected with the pro-cathepsin D cDNA (CD^−/−^CatD) were incubated for 14 h with 100 ng/ml TNF in combination with 20 μM zVAD-fmk and 5 μg/ml CHX or with 1 mM MNNG for 30 min and further incubated for 16 h with fresh medium without MNNG before loss of membrane integrity was determined

To address the role of the PARP pathway in TNF-induced necroptosis in more detail, we analyzed the impact of pharmacological inhibitors of PARP-1. The PARP-1 inhibitor 3-AB [25, 26] impaired activation of PARP-1 (i.e., the disappearance of the full-length PARP-1 signal) in all L929 sublines in response to TNF and MNNG (Fig. 3a). However, while 3-AB impaired MNNG-induced ATP depletion and necrosis, TNF-induced loss of ATP and necroptosis were unaffected in all three L929 cell lines (Fig. 3b). Similarly, the translocation of AIF into the cytosol of MNNG-treated cells was blocked by 3-AB, whereas the late TNF-induced AIF translocation remained unaltered in all L929 sublines (Fig. 3c). Thus, the inhibition of PARP-1 by 3-AB is not causally linked to TNF-induced necroptosis, and therefore does not prevent the secondary loss of ATP and translocation of AIF that occur in the course of cellular disintegration.

We extended our investigations to Jurkat and HT-29 cells, including murine NIH3T3 fibroblasts as a further cell systems for which TNF-induced necroptosis has been reported [7, 17]. In addition, we employed olaparib as a more potent and selective inhibitor of PARP-1 [26], and also the PARP-1 inhibitor PJ-34. As shown in Fig. 3d, all PARP-1 inhibitors (left panels) but not zVAD-fmk (right panel) effectively interfered with MNNG-induced necrosis. However, neither PARP-1 inhibitor reduced TNF(/zVAD/CHX)-induced necroptosis in any of the four cell lines (left panels). As the only exception, PJ-34 reduced TNF-mediated necroptosis in L929Ts cells. However, this was not observed in any of the other cell lines or with any of the other PARP-1 inhibitors and is therefore most likely due to unspecific activities that have been recently reported for PJ-34 [30, 31]. We obtained identical results after downregulation of PARP-1 in murine L929Ts and human Jurkat cells as well as in PARP-1-deficient MEF whereas MNNG-induced necrosis was clearly blocked (Fig. 3e).

JNK have been described as components of the PARP pathway required for mitochondrial dysfunction, AIF translocation, and necrosis [14]. However, pharmacological inhibition of JNK did not affect TNF-induced necroptosis in L929Ts cells, even at concentrations at which the inhibitor itself induced toxicity (Fig. 3f). In a direct genetic approach, neither primary lung fibroblasts from JNK1- or JNK2-deficient nor immortalized MEF from JNK1/JNK2 double-deficient mice showed reduced sensitivities to TNF-induced necroptosis relative to their respective wild-type littermate controls (Fig. 3g), dissimilar to the previously reported increased survival of JNK1^−/−^ and JNK2^−/−^ MEF in response to MNNG [14]. Furthermore, the RIP1 inhibitor necrostatin-1 failed to inhibit TNF-induced JNK activation in L929Ts cells, although necroptosis in response to TNF was completely blocked (Fig. 3h). Therefore, JNK do not participate in TNF-induced necroptosis, in contrast to their reported function in the PARP pathway [14].

As further constituents of the PARP pathway, calpain and cathepsin proteases cleave AIF to initiate its mitochondrio-nuclear translocation [13]. In TNF-induced necroptosis, caspase inhibitors, such as the broad-spectrum caspase inhibitor zVAD, but not individual application of inhibitors for calpain, cathepsin B, L, S, papain and cystein proteases potentiate the death response [17, 19, 23]. Even when combined, the cathepsin B/L inhibitor zFA-fmk, the cathepsin B inhibitor CA-074 Me and the broad-spectrum calpain/cysteine protease inhibitor E-64 failed to enhance TNF-induced necroptosis in L929Ts cells (Fig. 3i). Moreover, they failed to protect from the enhanced necroptotic response elicited by TNF/zVAD (Fig. 3i), suggesting that calpains, cathepsins B and L and cysteine proteases neither positively nor negatively participate in TNF-induced necroptosis. In controls, addition of zFA-fmk, E-64 and CA-074Me reduced MNNG-induced necrosis whereas zVAD-fmk had no effect (Fig. 3i), confirming the reported role of calpains and cathepsins in the PARP pathway. Furthermore, pharmacological inhibition of cathepsin D by pepstatin A (Fig. 3j) or analysis of wild-type versus cathepsin D-deficient or cathepsin D-deficient MEF ectopically re-expressing cathepsin D (Fig. 3k) revealed no differences in the necroptotic response to TNF, in summary demonstrating that despite their reported role in the PARP pathway, calpain and cathepsin proteases are not required for TNF-induced necroptosis. In control reactions, although pharmacological inhibition of cathepsin D indeed reduced MNNG-elicited necrosis in L929Ts, L929ATCC and L929sA cells (Fig. 3j), cathepsin D-deficient MEF were not protected from MNNG-induced necrosis in comparison to their wild-type counterparts or to cathepsin D-deficient MEF ectopically re-expressing cathepsin D (Fig. 3k). These results suggest that pepstatin A is protective in the PARP pathway by targeting proteases other than cathepsin D and that cathepsin D plays no role in either TNF-induced necroptosis or the PARP pathway. Altogether, the above data suggest that TNF-induced necroptosis is not affected by inhibition of PARP-1 or subsequent components of the PARP pathway.

### Interference with TNF-induced necroptosis does not block the PARP pathway

**Fig. 4:**
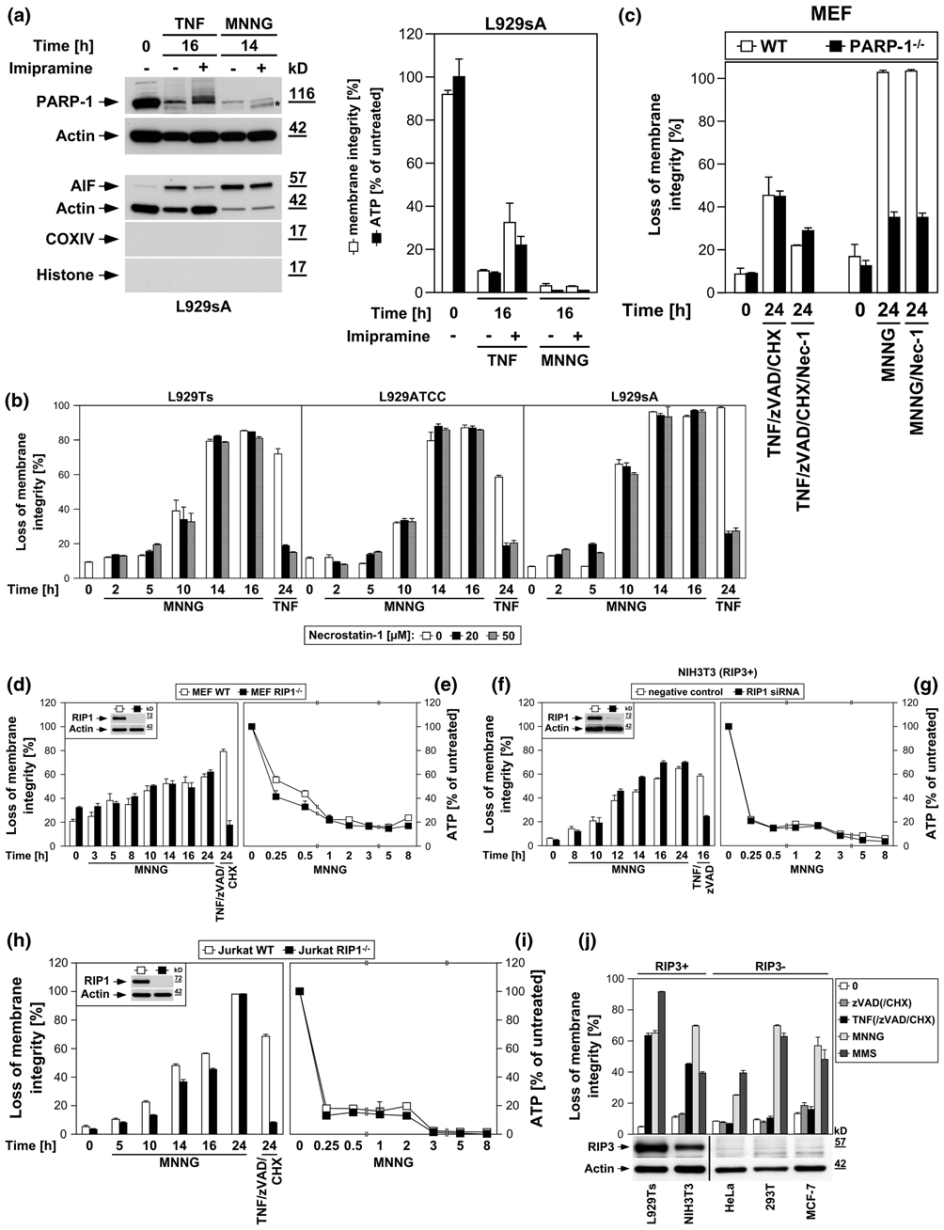

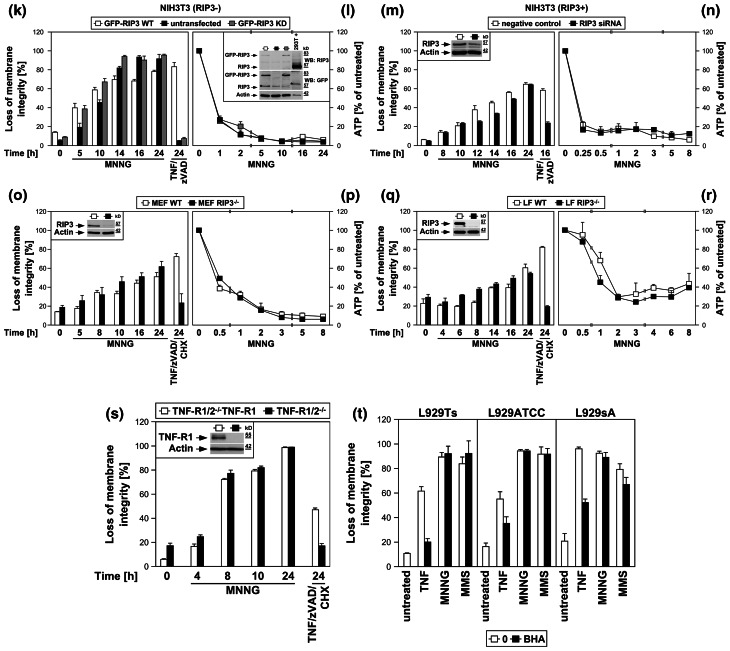
The PARP pathway is not blocked by interference with key mediators of TNF-induced necroptosis. **a** In the presence or absence of 25 μM imipramine, L929sA cells were stimulated with 100 ng/ml TNF for 0 or 16 h. Alternatively, the cells were treated with 1 mM MNNG for 15 min and further incubated with fresh medium without MNNG for the indicated times. PARP-1 was detected in Western blots with antibody 9542. Detection of actin served as a loading control. The *asterisk* indicates a nonspecific band. In parallel, nuclear translocation of AIF was determined using purified cytosolic nuclear fractions from the same experiment. Actin (cytosolic marker), COXIV (mitochondrial marker) and histone (nuclear marker) were used to control fractionation quality. Equal loading was verified by staining with Ponceau S (Supplementary Figure S2c), since actin displayed a decrease in these highly purified fractions during cell death. Imipramine selectively prevented this decrease as well as AIF translocation and activation/disappearance of PARP-1 in TNF-, but not MNNG-treated cells. In addition, intracellular ATP levels were measured and loss of membrane integrity was determined by flow cytometry and PI staining. For clarity of presentation, the percentage of intact, large PI-negative cells with preserved membrane integrity is shown. **b** L929Ts, L929ATCC and L929sA cells were treated with 100 ng/ml TNF for 24 h or with 0.5 mM MNNG for 15 min and further incubated for the indicated times with fresh medium without MNNG in the presence of the indicated concentrations of the RIP1 inhibitor necrostatin-1. Following stimulation, loss of membrane integrity was analyzed by flow cytometry. Necrostatin-1 clearly protected from TNF-induced necroptosis in all three L929 sublines at 20 μM, but not from MNNG-induced necrosis, even at a concentration of 50 μM. **c** Wild-type (WT) and PARP-1-deficient MEF were stimulated either with 0.5 mM MNNG for 15 min and further incubated with fresh medium without MNNG or with 100 ng/ml TNF, 20 μM zVAD-fmk and 2 μg/ml CHX in the presence or absence of 50 μM necrostatin-1 (Nec-1) for 24 h before loss of membrane integrity was measured by PI staining and flow cytometry. Necrostatin-1 reduced the TNF/zVAD/CHX-induced death of both wild-type and PARP-1-deficient MEF, indicating that TNF/zVAD/CHX indeed elicits necroptosis in these cells. **d** Wild-type (WT) and RIP1-deficient (RIP1^−/−^) MEF were stimulated for the indicated times with MNNG as in (**b**) or with 100 ng/ml TNF in combination with 20 μM zVAD-fmk and 2 μg/ml CHX before loss of membrane integrity was measured by flow cytometry. *Inset* control Western blot for murine RIP1 or actin to verify presence or absence of endogenous murine RIP1 and equal loading. **e** Intracellular ATP levels were analyzed from the same cells stimulated with MNNG for the indicated times as in (**b**). **f**, **g** NIH3T3 cells naturally expressing RIP3 were nucleofected with an siRNA that does not elicit an RNA interference response (negative control) or with siRNA specific for murine RIP1 (RIP1 siRNA) and analyzed as in (**d**, **e**), except that CHX was omitted from treatment. *Inset* control Western blot for murine RIP1 to verify successful downregulation. Detection of actin served as a loading control. **h**, **i** Wild-type (WT) and RIP1-deficient (RIP1^−/−^) Jurkat cells were analyzed as in (**d**, **e**), except that 50 μM zVAD-fmk and 2 μg/ml CHX were used for treatment with TNF/zVAD/CHX. *Inset* control Western blot for human RIP1 or actin to verify presence or absence of endogenous human RIP1 and equal loading. *p* values in (**h**) calculated using Student’s *t* test for WT versus RIP^−/−^ cells were <0.001 in all instances (except for 24 h of MNNG treatment). **j** RIP3-positive or -negative cells were treated with TNF for 24 h or with MNNG (or 25 mM MMS) for 16 h as in (**b**) with addition of 20 μM (NIH3T3) or 50 μM (293T, MCF-7, HeLa) zVAD-fmk and 1 ng/ml (293T) or 0.25 μg/ml (MCF-7, HeLa) CHX before loss of membrane integrity was determined flow-cytometrically. Controls were included to demonstrate that treatment with zVAD-fmk(/CHX) alone was not toxic. The presence or absence of RIP3 was verified by Western blots with antibodies specific for murine (L929, NIH3T3) or human RIP3 (HeLa, 293T, MCF-7). The specificity of the antibodies was confirmed with cell lysates containing overexpressed murine or human RIP3 (not shown). Equal loading was verified by detection of actin. **k**, **l** NIH3T3 cells naturally deficient for RIP3 or stably retransfected with wild-type or kinase-defective murine GFP-RIP3 (GFP-RIP3 WT, GFP-RIP3 KD) were treated with MNNG or TNF/zVAD and analyzed as in (**f**, **g**). *Inset* control Western blot for murine RIP3 (*top*), GFP (*middle*) or actin (*bottom*) to verify expression of the constructs, absence of endogenous murine RIP3, and equal loading. A reduced amount of lysate from 293T cells overexpressing untagged murine RIP3 was used as control (293T+). *Asterisks* nonspecific bands. *p* values in (**k**) calculated using Student’s *t* test for GFP-RIP3 WT versus untransfected NIH3T3 cells were <0.001 for 0, 5 and 10 h of treatment with MNNG and 24 h of treatment with TNF/zVAD. **m**, **n** As part of the same experiment as shown in (**f**, **g**), NIH3T3 cells naturally expressing RIP3 were additionally nucleofected with siRNAs specific for murine RIP3 and analyzed as in (**f**, **g**). For clarity of presentation, the data are shown in new panels, but with the same negative control values as in (**f**, **g**). *Inset* control Western blot for downregulation of murine RIP3. Actin was detected as a loading control. Except for 24 h of MNNG treatment, *p* values in (**m**) calculated using Student’s *t* test for cells transfected with negative control siRNA versus RIP3 siRNA were <0.01 (0 and 12 h of MNNG treatment) or <0.001 (all other time points). **o**, **p** Primary MEF or **q**, **r** primary lung fibroblasts (LF) from wild-type (WT) or RIP3-deficient (RIP3^−/−^) mice were analyzed as in (**f**, **g**). *Insets* control Western blots for murine RIP3 or actin to verify presence or absence of endogenous murine RIP3 and equal loading. **s** TNF-R1/TNF-R2 double-deficient murine fibroblasts (TNF-R1/2^−/−^) and TNF-R1/2^−/−^-fibroblasts stably re-expressing TNF-R1 (TNF-R1/2^−/−^TNF-R1) were analyzed as in (**d**). *Inset* control Western blot for expression or absence of TNF-R1. Actin was detected as a loading control. **t** L929Ts, L929ATCC and L929sA cells were treated with 100 ng/ml TNF for 24 h or with MNNG (0.5 mM) or MMS (25 mM) for 15 min and further incubated for 16 h with fresh medium without MNNG or MMS, with or without addition of the radical scavenger BHA (150 μM, 2 h pretreatment). Subsequently, loss of membrane integrity was analyzed by flow cytometry

In a reverse approach, we examined whether interference with known key mediators of TNF-induced necroptosis also affected necrosis through the PARP pathway. The antidepressant imipramine prevents accumulation of intracellular ceramide, a pivotal molecule in death receptor-elicited necroptosis [16, 17, 19]. In L929sA cells, imipramine impaired PARP-1 activation/disappearance, AIF translocation, cell death and the accompanying loss of ATP as late events in TNF-induced necroptosis but not in response to MNNG (Fig. 4a).

Inhibition of RIP1 by necrostatin-1 clearly protected from TNF-induced necroptosis in all three L929 sublines, but had no effect on the course of MNNG-induced necrosis over 16 h (Fig. 4b). Likewise, TNF/zVAD/CHX-induced necroptosis was reduced by necrostatin-1 in both wild-type and PARP-1-deficient MEF, whereas their differential response towards MNNG-induced necrosis remained completely unaffected (Fig. 4c). RIP1-deficient MEF were fully resistant to TNF/zVAD/CHX-induced necroptosis, but not noticeably protected from MNNG (Fig. 4d). Downregulation of RIP1 in NIH3T3 cells (that naturally express RIP3) [7], protected from TNF/zVAD-induced necroptosis, but not from MNNG (Fig. 4f). RIP1-deficient Jurkat cells proved fully resistant against TNF/zVAD/CHX-induced necroptosis, but, other than MEF and NIH3T3 cells, displayed a transient, statistically significant protection from MNNG, however not preventing final death (Fig. 4h). Absence or downregulation of RIP1 did not prevent the MNNG-induced rapid loss of intracellular ATP as an early step of the PARP pathway (Figs. 4e, g, i), in line with previous studies suggesting that RIP1 may also function in the PARP pathway, however at later stages [14, 27] rather than in the central, initial steps (as in TNF-induced necroptosis).

In line with the crucial function of RIP3 in necroptosis [6, 7, 10], TNF(/zVAD/CHX) elicited necroptosis in L929Ts and NIH3T3 cells expressing RIP3, but not in 293T, MCF-7 and HeLa cells that lack endogenous RIP3 expression. In contrast, both MNNG and MMS uniformly induced necrosis in all cell lines (Fig. 4j). To exclude artifacts from these different constitutively RIP3-positive or -negative cell lines, we overexpressed wild-type or kinase-defective RIP3 in naturally RIP3-deficient NIH3T3 cells [7] and downregulated RIP3 in NIH3T3 cells that naturally express RIP3 [7]. Similar to RIP1-deficient Jurkat cells (Fig. 4h), RIP3-deficient NIH3T3 cells displayed a statistically significant transient attenuation, but not protection from MNNG, while being fully resistant against TNF/zVAD-induced necroptosis (Fig. 4k). Stable re-expression of wild-type, but not kinase-defective RIP3 restored full sensitivity to TNF/zVAD [6, 10]. In contrast, sensitivity for MNNG was restored by re-expression of both wild-type and kinase-defective RIP3, indicating that the kinase activity of RIP3 is not required for its function in the PARP pathway. In reverse, downregulation of RIP3 protected naturally RIP3-expressing NIH3T3 cells from TNF/zVAD-induced necroptosis while only transiently (but statistically significant) attenuating MNNG-induced necrosis (Fig. 4m). In primary MEF or primary lung fibroblasts from RIP3-deficient mice, TNF/zVAD/CHX-induced necroptosis was completely blocked, whereas the course of MNNG-induced necrosis was not altered (Fig. 4o, q). As for RIP1, the rapid loss of ATP during MNNG-induced necrosis occurred identically in RIP3-deficient, -downregulated or -retransfected cells (Fig. 4l, n, p, r), implicating that RIP3 also does not participate in the initial but later steps of the PARP pathway.

Consistent with the above studies [14, 27], our data suggest a function of RIP1 in both TNF-induced necroptosis and MNNG-mediated necrosis. However, while RIP1 is clearly crucial for TNF-induced necroptosis in a cell type-independent manner, its contribution to MNNG-induced necrosis appears to be of more limited importance and cell type-specific. Although previously not investigated, our data indicate that the same is also true for RIP3. Notably, Xu et al. [14] have previously ruled out that the function of RIP1 in MNNG-induced necrosis depends on the TNF signaling pathway by demonstrating that MEF deficient for TNF-R1 remain as sensitive to MNNG as wild-type MEF. We extended this experiment by showing that the course of MNNG-induced necrosis was identical in both TNF-R1/TNF-R2 double-deficient fibroblasts and TNF-R1/TNF-R2 double-deficient control fibroblasts that ectopically re-express TNF-R1, thus clearly demonstrating that any role of RIP1 or RIP3 in the PARP pathway cannot depend on signals mediated by TNF-R1 (Fig. 4s). In combination with the kinase-independent function of RIP3 in the PARP pathway (contrasting TNF-dependent necroptosis), our results demonstrate that although RIP1 and RIP3 participate in both TNF-induced necroptosis and the PARP pathway, their functions in each pathway are independent and do not connect the two pathways.

Finally, scavengers of ROS such as BHA can protect L929 cells from TNF-induced necroptosis [17, 23]. Correspondingly, BHA decreased necroptosis in TNF-treated L929Ts, L929ATCC and L929sA cells, but failed to protect against necrosis induced by MNNG and MMS in any of the three cell lines (Fig. 4t).

**Fig. 5:**
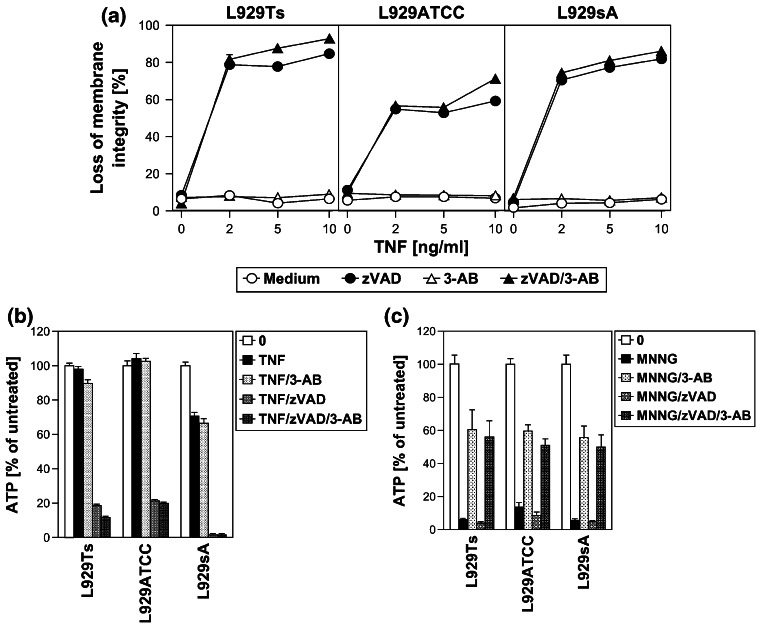
The PARP-1 inhibitor 3-AB does not counteract TNF-induced loss of membrane integrity and ATP depletion in L929 cells. **a** L929Ts, L929ATCC and L929sA cells were preincubated for 2 h with 40 μM zVAD-fmk, 3 mM 3-AB, or a combination of both, and then treated with the indicated concentrations of TNF for 5 h before loss of membrane integrity was determined flow-cytometrically. **b** Cells were preincubated and treated with 3-AB and/or zVAD-fmk as in (**a**) and stimulated with 40 ng/ml TNF for 4 h before intracellular ATP levels were determined. **c** To verify the efficacy of 3-AB under the conditions used above, L929Ts, L929ATCC and L929sA cells were preincubated for 2 h with 40 μM zVAD-fmk, 3 mM 3-AB, or a combination of both, and then with 0.5 mM MNNG for 16 h before ATP depletion was determined

In contrast to the results obtained here, a previous study has suggested an important role of the PARP pathway in TNF-induced necroptosis of L929 cells [15]. We therefore replicated the key experiments of the above study (protective effects of the PARP-1 inhibitor 3-AB against TNF- or TNF/zVAD-induced necroptosis and against loss of intracellular ATP) under exactly the same experimental conditions for L929Ts, L929ATCC and L929sA cells. Fully consistent with our above findings, 3-AB did not prevent necroptosis measured by loss of membrane integrity (Fig. 5a) or by loss of ATP (Fig. 5b) in any of the three cell lines (but effectively interfered with MNNG-induced necrosis in controls, Fig. 5c). As a likely explanation, Los et al. utilized a single transfectant clone of L929 cells stably expressing CD95 (L929-APO-1-6) for their experiments [15, 32], which may show altered responses in comparison to the parental, untransfected L929 cells lines used in this study.

## Discussion

In this study, we present evidence that TNF-induced necroptosis and the PARP pathway represent two distinct and independent routes to programmed necrosis. The importance of programmed necrosis, especially in pathological situations, becomes increasingly obvious and the possibility for therapeutic suppression of necrosis has raised great expectations [1]. A role in the pathology of diseases has been reported for both necroptosis and PARP-1-dependent necrosis. Inhibition of necroptosis by targeting RIP1 effectively reduced renal [8], cardiac and retinal ischemia/reperfusion injury [1], limited brain damage, and improved motor and cognitive performance in mouse models [1]. Similarly, genetic deficiency for RIP3 limited tissue damage in a pancreatitis model [1]. PARP-1-dependent necrosis plays a central role in inflammatory responses in cells of the central nervous, cardiovascular, and immune systems. PARP-1 inhibitors have been effective in the treatment of ischemia/reperfusion damage after stroke or brain, myocardial, hepatic, and renal ischemia/reperfusion injury, other acute forms of cardiomyopathies and heart failure, tissue injury in response to septic and hemorrhagic shock, as well as acute lung inflammation, peritonitis, and pancreatitis. Inhibition of the PARP pathway has also demonstrated benefit in chronic disease models, e.g., cardiovascular aging, atherosclerosis, and diabetic cardiovascular complications. Furthermore, the PARP pathway represents a promising target for treatment of autoimmune conditions, including type 1 diabetes, glomerulonephritis, multiple sclerosis, and rheumatoid arthritis [1, 25, 33].

**Fig. 6:**
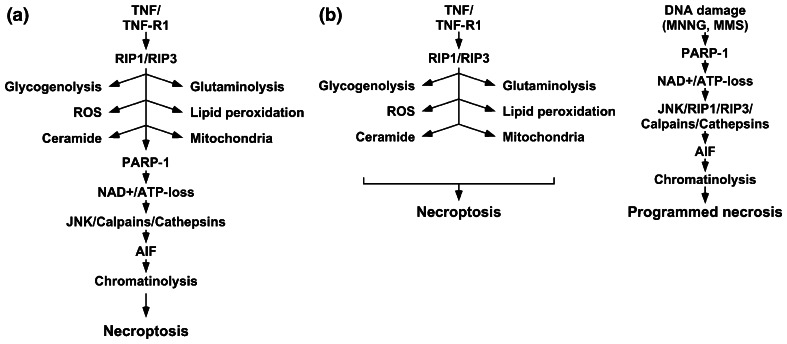
Previous and revised model of the PARP pathway as a component of necroptosis. **a** In the previously established model, the PARP pathway represents an integral component of necroptosis. In this model, binding of TNF to TNF-R1 induces activation of RIP1/RIP3, and subsequently triggers a panel of effector mechanisms such as glycogenolysis, glutaminolysis, formation of ROS, lipid peroxidation, formation of ceramide, and mitochondrial dysfunction. As a central element, the overactivation of PARP-1 causes depletion of NAD^+^/ATP, activation of JNK, calpains and cathepsins, mitochondrio-nuclear translocation of AIF, chromatinolysis, and finally, necroptosis. **b** In the revised model suggested in this study, the PARP pathway is activated, e.g., in response to DNA damage and mediates programmed necrosis independently from TNF-induced necroptosis. Although RIP1 and RIP3 participate in both pathways, they fulfill nonredundant, independent functions and do not interconnect the two pathways

In the model currently established for L929 cells (Fig. 6a), the PARP pathway represents an integral part of TNF-induced necroptosis [1, 15]. The results obtained in our study in multiple cell systems, however, clearly indicate that this is not the case. Rather, TNF-induced necroptosis and PARP-1-mediated necrosis apparently operate independently and represent separate routes to programmed necrosis. Of note, although our data show for the first time that RIP3 and, in extension of previous reports [14, 27], also RIP1 indeed do participate in the PARP pathway, our results nevertheless demonstrate that this contribution is cell type-specific and only of limited impact, in contrast to the ubiquitous, essential function of RIP1 and RIP3 in TNF-induced necroptosis. Most importantly, we have demonstrated that any role of RIP1 or RIP3 in the PARP pathway cannot depend on signals mediated by TNF-R1, and thus revalidated our concept of TNF-induced necroptosis and PARP-1-mediated necrosis as distinct and independent routes to programmed necrotic cell death. This revised model (Fig. 6b) is additionally corroborated by data from independent studies. Seok et al. [34] demonstrated that JNK activation by DNA-damaging agents occurs in a TNF-independent manner, although RIP1 is required. Two independent studies [35, 36] failed to find an obligate role of JNK in TNF-induced cell death in multiple cell systems, despite their reported role in the PARP pathway [14]. Identical to our results, induction of necroptosis in L929 cells with TNF did not lead to detectable AIF release within 12 h [37]. In Jurkat and BALB/c 3T3 cells, necroptotic cell death could not be blocked by small-molecule inhibition of factors such as calpains and PARP-1, or the downregulation of AIF by RNA interference [38]. Inhibition of cathepsins B, L, H, or D as further components of the PARP pathway [13] failed to affect TNF-induced necroptosis in murine hepatocytes [39]. Conversely, necrostatin-1 did not protect murine hippocampal HT-22 cells against MNNG-induced necrosis [40]. Andrabi et al. [41] independently found that necroptosis-resistant HeLa cells are still sensitive to MNNG-induced necrosis, and the failure of BHA to protect against MNNG has been reported also for Chinese hamster V79 cells [42].

Notably, Jouan-Lanhouet et al. [43] have recently reported that TRAIL induces necroptosis at acidic extracellular pH (pHe) involving RIP1/RIP3-dependent PARP-1 activation, seeming to be at odds with our results at first glance. However, the same group has previously reported that in spite of a necrosis-like cell death morphology, caspase activity is required for cell death in this system and that both zVAD-fmk and the caspase-8 inhibitor zIETD-fmk fully block TRAIL-induced cell death at acidic pHe [44]. This dependence on caspases is however not consistent with the molecular mechanisms described for necroptosis [45] but rather points to a mode of cell death different from TNF-induced necroptosis studied here, where we have never observed an inhibitory effect of zVAD-fmk in the many experiments where it was added. Therefore, an involvement of PARP-1 in the TRAIL-induced mode of cell death described by Jouan-Lanhouet is feasible based on the data presented in their study, but does not implicate a similar role of PARP-1 in TNF-induced necroptosis as studied here. Also, such a role still would have to be validated for true necroptosis induced by TRAIL, e.g., in the presence of zVAD-fmk, and at physiologic pHe (7.4). Moreover, the effects described in the study of Jouan-Lanhouet et al. do not occur at physiologic pHe but are exclusively limited to acidic conditions, again different from our observations for TNF-induced necroptosis. Finally, Jouan-Lanhouet et al. [43] themselves demonstrate that their findings do not apply to necroptosis induced by either TNF or FasL, regardless of physiologic or acidic pHe, again suggesting that the conclusions from their study cannot be easily generalized for other death receptors or physiologic conditions.

Of particular importance, this study provides a first answer to the recently raised question whether there is one core program or several independent pathways of necrosis, with implications for the future development of necroptosis-inhibitory cytoprotective drugs as well as for the design of therapeutic strategies. The existence of several distinct pathways that trigger necrosis implies that an efficient protection requires their simultaneous interruption via combination therapies [1]. As a consequence of the results obtained in this study, we suggest that the simultaneous rather than the individual application of highly specific necroptosis and PARP-inhibitors (e.g., necrostatin and olaparib) may be the approach of choice for a future, improved treatment of necrosis-induced damage in diseases such as shock, stroke, or myocardial infarction.

### Electronic supplementary material

Below is the link to the electronic supplementary material.

Supplementary material 1 (PDF 5714 kb)
