# Supplementary material for: TNF-induced necroptosis and PARP-1-mediated necrosis represent distinct routes to programmed necrotic cell death
Source: Cell Mol Life Sci. 2013 Jun 13;71(2):331–48. doi: 10.1007/s00018-013-1381-6 (PMC3889832; doi:10.1007/s00018-013-1381-6)
Supplement: Supplementary file 1 — Supplementary material 1 (PDF 5714 kb) [file 18_2013_1381_MOESM1_ESM.pdf]

<u>Supplementary information</u>

Supplementary materials and methods

Measurement of caspase-3 activity. Following treatment, adherent and detached cells were collected and lysed in a buffer containing 10 mM Hepes pH 7.4, 142 mM KCl, 5 mM $MgCl_2$, 1 mM EGTA, 0.2% v/v NP40, 1 mM DTT and 2 mM Pefabloc (Roche, Mannheim, Germany). To measure caspase activity, 100 μl of caspase buffer (20 mM Pipes, 100 mM NaCl, 10 mM DTT, 1 mM EDTA, 0.1% w/v CHAPS, 10% w/v sucrose, pH 7.2) containing 100 μM zDEVD-afc (benzyloxycarbonyl-Asp(OMe)-Glu(OMe)-Val-DL-Asp(OMe)-7-aminotrifluoromethylcoumarin, Calbiochem, Bad Soden, Germany) were added to 10 μl of cytosolic extract (20 μg protein) and incubated at 37°C. The release of afc was measured as emission at 505 nm upon excitation at 405 nm using an Infinite M200 fluorimeter equipped with a thermostated plate reader (Tecan, Crailsheim, Germany).

Microscopy. For documentation of cell morphology, images from unfixed cells were obtained using an Axiovert 10 microscope (Zeiss, Oberkochen, Germany) and a DS-5M-L1 digital sight camera system (Nikon, Düsseldorf, Germany).

Ponceau S staining. Nitrocellulose membranes were removed from the electrophoretic transfer chamber, rinsed with Milli-Q water, incubated in a solution of 0.5% w/v Ponceau S in 1% v/v acetic acid for 10 min and rinsed again with Milli-Q water to remove background. Subsequently, efficiency of transfer and equality of loading was documented by scanning the membranes on an Epson Perfection 4180 Photo scanner (Epson, Meerbusch, Germany).

Supplementary Figure Legends

Fig. S1 TNF and MNNG do not elicit caspase-dependent apoptosis in L929 cells. (a) L929Ts, L929ATCC and L929sA cells were left untreated, treated with 100 ng/ml TNF for 5 h, or treated with 0.5 mM MNNG for 15 min and further incubated with fresh medium without MNNG for another 5 h. As a positive control, the cells were treated with 100 ng/ml TNF and 2 μg/ml CHX for 5 h. Subsequently, activation of caspase-3 as an indicator for caspase-dependent apoptosis through both the extrinsic and the intrinsic pathway was determined by measuring the cleavage of the fluorogenic substrate zDEVD-afc over 120 minutes. (b) Micrographs showing the morphology of L929Ts, L929ATCC or L929sA cells stimulated as in (a) for the indicated times. Black arrows indicate cells that show apoptotic membrane blebbing in response to TNF/CHX. White arrows indicate the distinct necrosis-like changes in cells after treatment with TNF or MNNG. For both TNF and MNNG, the timepoints shown depict cells where morphological alterations are not yet detectable, followed by the earliest timepoint where changes in cell morphology became apparent, followed by a later timepoint where the morphological alterations are pronouncedly visible. Scale bar: 100 μM

Fig. S2 Controls for equal loading by staining with Ponceau S and for activity of the utilized COXIV and histone antibodies. (a) Ponceau S staining of the membranes corresponding to the blots shown in Fig. 2d, (b) in Fig. 3c, or (c) in the lower panels of Fig. 4a. (d) The unstimulated lysates from L929Ts, L929ATCC and L929sA cells that are shown in Fig. 2d were taken as representative examples, reloaded aside with positive control lysates (Co) for COXIV (mouse brain extract, Santa Cruz, sc-2253) and histone (HeLa cell lysate) and analyzed as in Fig. 2d to confirm the activity of the utilized COXIV and histone antibodies

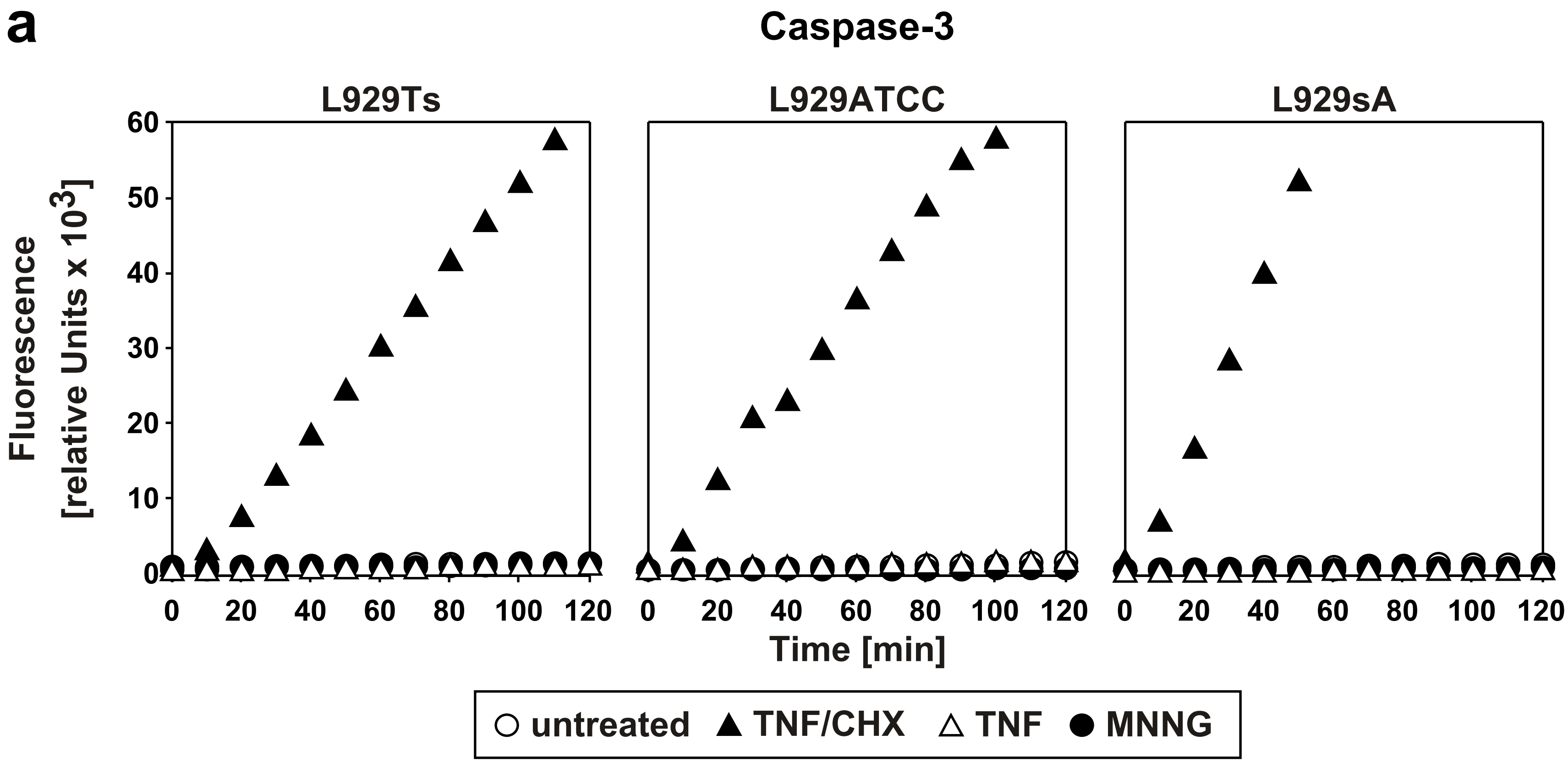
a
Caspase-3
L929Ts
L929ATCC
L929sA
Fluorescence
[relative Units x 10³]
0
10
20
30
40
50
60
0 20 40 60 80 100 120
Time [min]
untreated
TNF/CHX
TNF
MNNG

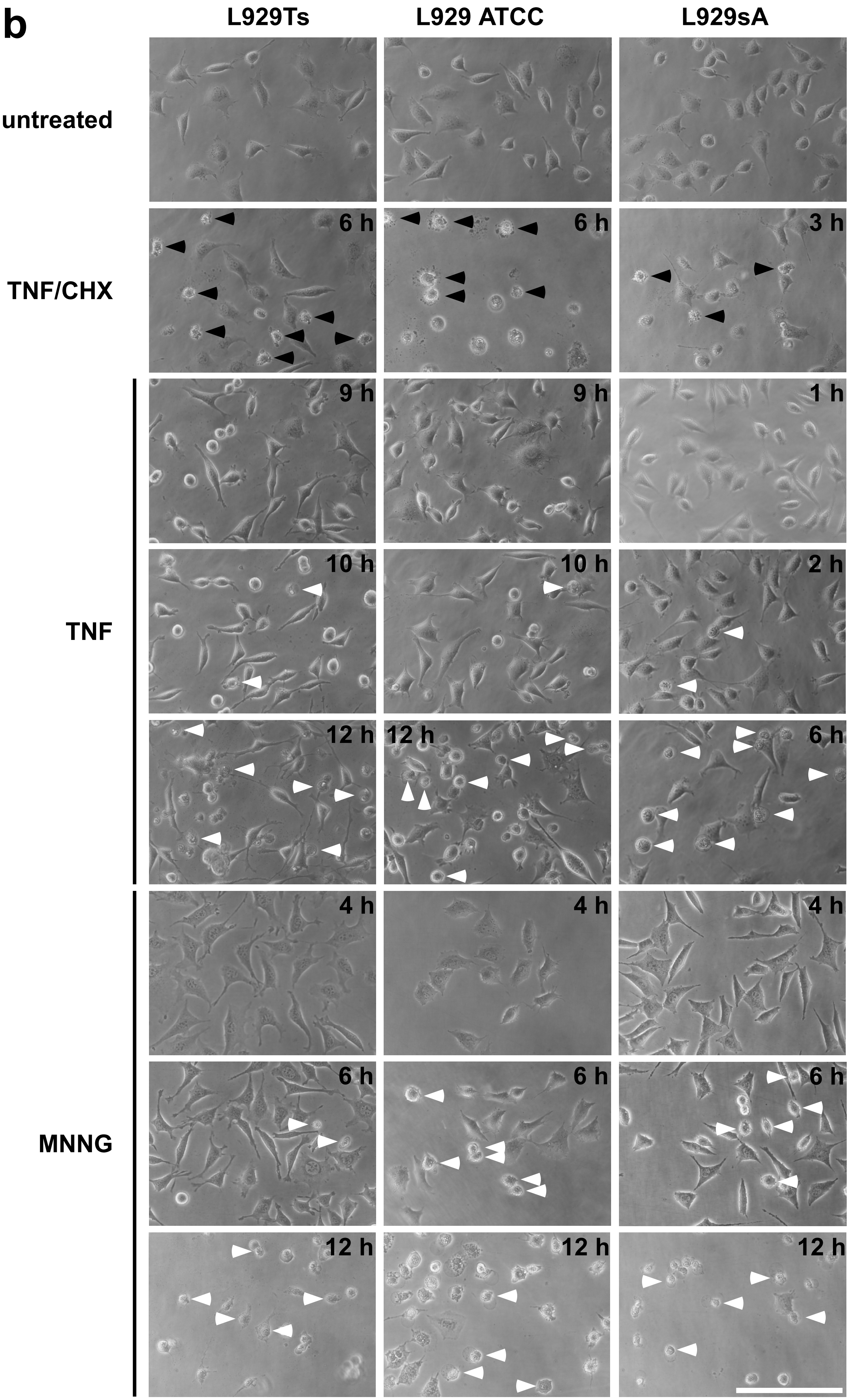
b
L929Ts
L929 ATCC
L929sA
untreated
TNF/CHX
TNF
MNNG
6 h
3 h
9 h
1 h
10 h
2 h
12 h
6 h
4 h
6 h
12 h

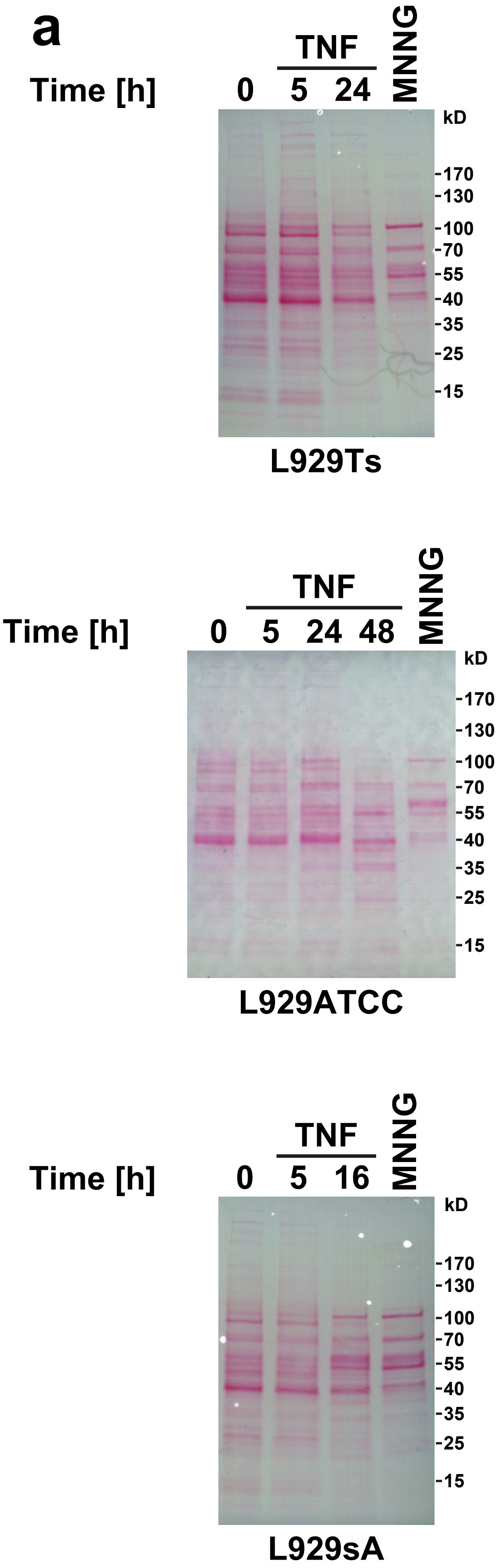
a
Time [h]
TNF
0
5
24
MNNG
kD
-170
-130
-100
-70
-55
-40
-35
-25
-15
L929Ts
Time [h]
TNF
0
5
24
48
MNNG
kD
-170
-130
-100
-70
-55
-40
-35
-25
-15
L929ATCC
Time [h]
TNF
0
5
16
MNNG
kD
-170
-130
-100
-70
-55
-40
-35
-25
-15
L929sA

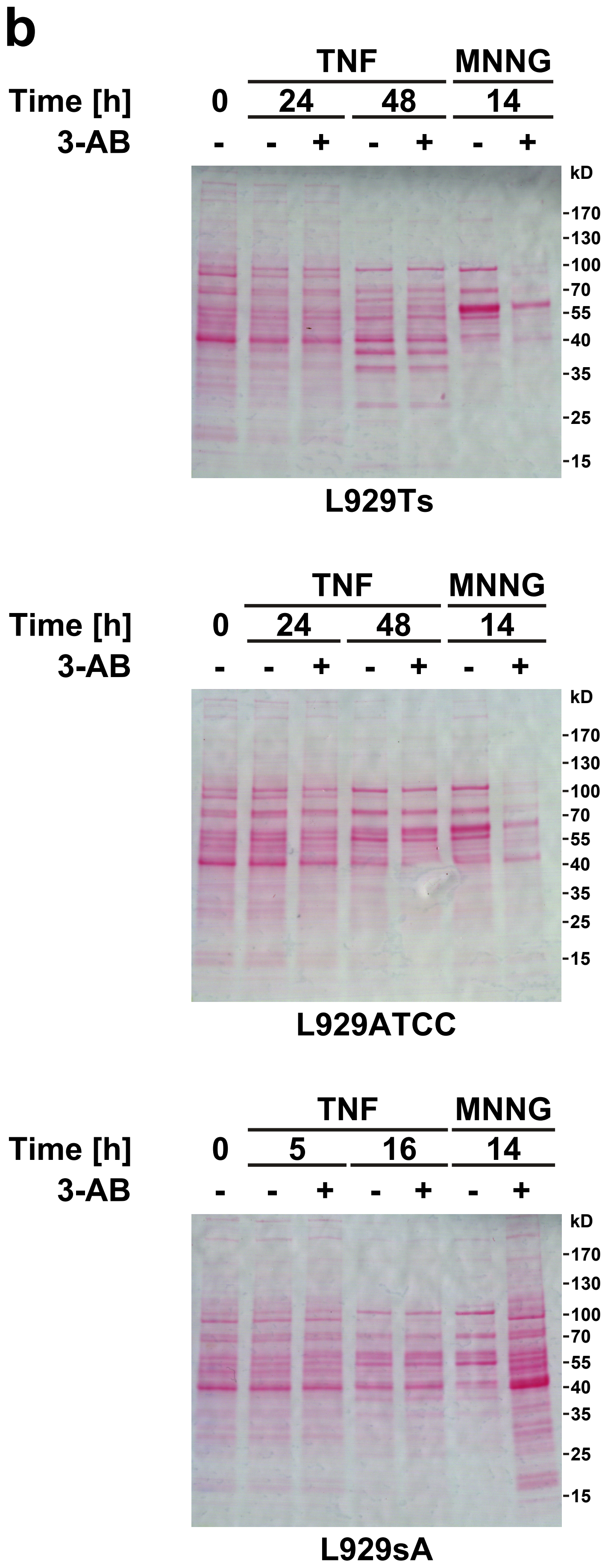
b
TNF
MNNG
Time [h]
0
24
48
14
3-AB
- - + - + - +
kD
-170
-130
-100
-70
-55
-40
-35
-25
-15
L929Ts
TNF
MNNG
Time [h]
0
24
48
14
3-AB
- - + - + - +
kD
-170
-130
-100
-70
-55
-40
-35
-25
-15
L929ATCC
TNF
MNNG
Time [h]
0
5
16
14
3-AB
- - + - + - +
kD
-170
-130
-100
-70
-55
-40
-35
-25
-15
L929sA

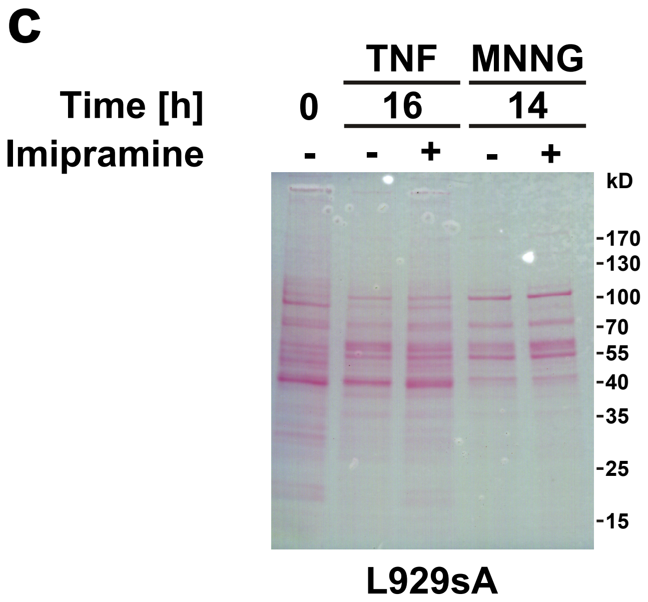
c
TNF
MNNG
Time [h]
0
16
14
Imipramine
-
-
+
-
+
kD
-170
-130
-100
-70
-55
-40
-35
-25
-15
L929sA

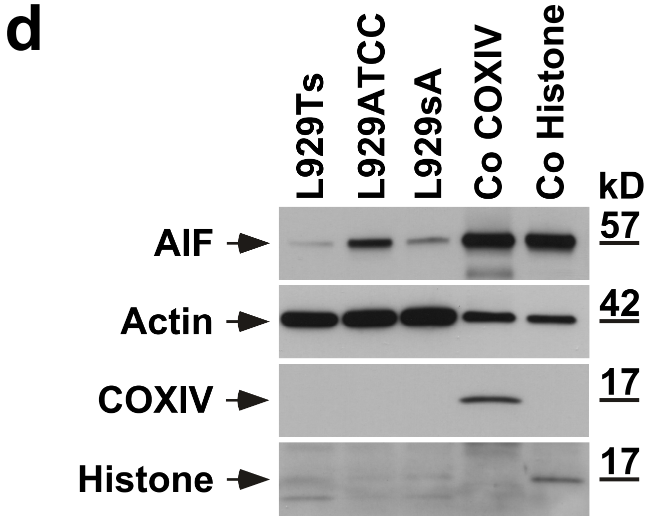
d
L929Ts
L929ATCC
L929sA
Co COXIV
Co Histone
kD
AIF
57
Actin
42
COXIV
17
Histone
17
